# TDP-43 or FUS-induced misfolded human wild-type SOD1 can propagate intercellularly in a prion-like fashion

**DOI:** 10.1038/srep22155

**Published:** 2016-03-01

**Authors:** Edward Pokrishevsky, Leslie I. Grad, Neil R. Cashman

**Affiliations:** 1 Centre for Brain Health, University of British Columbia, Vancouver, Canada

## Abstract

Amyotrophic lateral sclerosis (ALS), which appears to spread through the neuroaxis in a spatiotemporally restricted manner, is linked to heritable mutations in genes encoding SOD1, TDP-43, FUS, C9ORF72, or can occur sporadically without recognized genetic mutations. Misfolded human wild-type (HuWt) SOD1 has been detected in both familial and sporadic ALS patients, despite mutations in SOD1 accounting for only 2% of total cases. We previously showed that accumulation of pathological TDP-43 or FUS coexist with misfolded HuWtSOD1 in patient motor neurons, and can trigger its misfolding in cultured cells. Here, we used immunocytochemistry and immunoprecipitation to demonstrate that TDP-43 or FUS-induced misfolded HuWtSOD1 can propagate from cell-to-cell via conditioned media, and seed cytotoxic misfolding of endogenous HuWtSOD1 in the recipient cells in a prion-like fashion. Knockdown of SOD1 using siRNA in recipient cells, or incubation of conditioned media with misfolded SOD1-specific antibodies, inhibits intercellular transmission, indicating that HuWtSOD1 is an obligate seed and substrate of propagated misfolding. In this system, intercellular spread of SOD1 misfolding is not accompanied by transmission of TDP-43 or FUS pathology. Our findings argue that pathological TDP-43 and FUS may exert motor neuron pathology in ALS through the initiation of propagated misfolding of SOD1.

Amyotrophic lateral sclerosis (ALS) is a fatal neurodegenerative disorder characterized by degeneration of both upper and lower motor neurons, leading to progressive paralysis in muscles of the limbs, speech, swallowing and respiration. Nearly 90% of ALS cases are sporadic (SALS) with no known Mendelian genetic component, while the remaining 10% of cases are hereditary in a primarily autosomal dominant fashion. In familial ALS (FALS), the presence of any one of over 180 inherited mutations in the gene that encodes Cu/Zn superoxide dismutase (SOD1; http://alsod.iop.kcl.ac.uk/), a cytosolic scavenger of the superoxide anion radical, can lead to misfolding of the protein and to its toxic gain of function^1^,^2^. Additionally, multiple studies have detected misfolded forms of human wild-type SOD1 (HuWtSOD1) protein in SALS and FALS in the absence of SOD1 mutations^3^,^4^,^5^, suggesting that non-native conformers of SOD1 may play a key pathological role in all cases of ALS. However, the presence of misfolded SOD1 in sporadic disease remains a controversial topic^6^,^7^ as not all conformation specific antibodies can equally detect the aberrant forms of wild-type SOD1 in sporadic disease, which could be attributed to the differing epitope specificity and affinity of the antibodies employed. The disease can also be caused by mutations in either TAR-DNA binding protein 43 (TDP-43)^8^,^9^, or fused in sarcoma (FUS; originally designated translocated in liposarcoma, TLS)^10^,^11^, as well as other proteins (reviewed in reference 12). Both TDP-43 and FUS are predominantly nuclear RNA binding proteins that are redistributed in a mutually exclusive fashion to the cytosol in FALS, where they form insoluble inclusions^8^,^13^,^14^. Furthermore, post-mortem immunohistochemistry shows that all known cases of SALS, as well as non-SOD1 and non-FUS-FALS, contain neuronal and glial wild-type (wt)TDP-43 inclusions^14^. The clinicopathological similarities among all types of ALS, as well as the co-presence of mislocalized TDP-43 or FUS along with misfolded SOD1 in pathology, led us to determine that aberrant cytoplasmic localization of TDP-43 or FUS triggers misfolding of HuWtSOD1 in cell culture models^4^.

ALS pathology may begin at a single focal or multifocal sites; however, disease appears to spread through the neuroaxis in a spatiotemporal manner^15^,^16^. This systematic spread of disease is consistent with a prion-like propagation of misfolded protein in disease, for which several protein candidates have been proposed in ALS. Insoluble TDP-43 from diseased brains has been reported to induce TDP-43 pathology in neuroblastoma cells that overexpress wtTDP-43 as detected by TDP-43 hyperphosphorylation, ubiquitination and aggregation^17^. SOD1 also can display prion-like properties: we have established a cell culture system in which endogenous HuWtSOD1 was induced to misfold in the presence of misfolded mutant SOD1 ‘seed’^18^. Subsequent studies demonstrated that misfolded HuWtSOD1 can be propagated intercellularly via exosome-dependent and independent mechanisms^19^. Thus, induced misfolding of HuWtSOD1 by mutant SOD1 constructs expressed via transient cell transfection acquires propagation competency, and the capability to confer its misfold indefinitely on subsequent cell culture passages, even after the initial mutant protein is no longer detectable due to dilution and degradation^20^. *In vivo* studies have also recently demonstrated the presence of paralysis-associated seeded aggregation of fluorescently tagged SOD1^G85R^ in neonatally inoculated mice^21^. Here, we asked whether TDP-43 or FUS-induced misfolded HuWtSOD1 also acquires the prion-like property of seeding HuWtSOD1 propagated misfolding that can be passaged from cell culture to cell culture via conditioned media.

## Results

### TDP-43 or FUS-induced misfolded HuWtSOD1 can propagate intercellularly and seed cytotoxic misfolding of endogenous HuWtSOD1 in recipient cells

**Figure 1:**
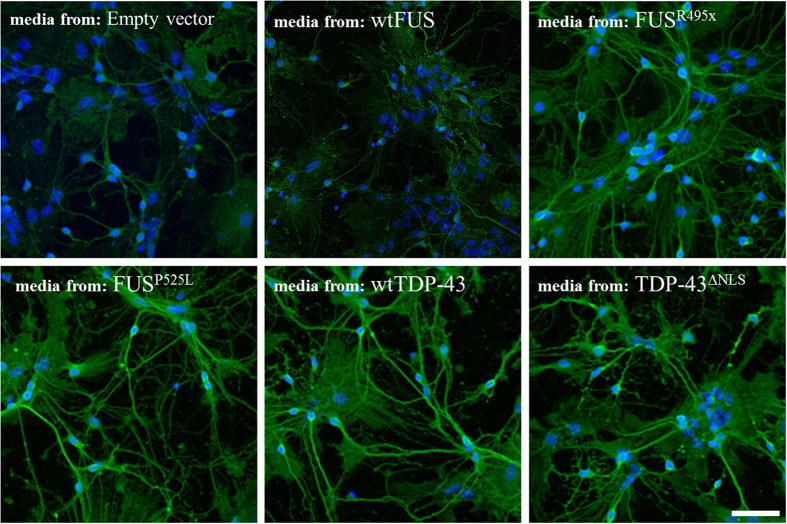
TDP-43 and FUS-induced misfolded HuWtSOD1 propagates from transfected cells to untreated spinal cord cultures. Primary spinal cord cultures containing neurons (including motor neurons) and astrocytes prepared from human HuWtSOD1 transgenic mice were incubated for 20 h with conditioned media from transfected HEK293 cells, and stained for misfolded SOD1 (green) using misfolded SOD1-specific antibody, 3H1, and counterstained using Hoechst 33342 (blue). The source of the media is indicated for each panel. Primary cultures incubated with conditioned media from FUS^R495X^, FUS^P525L^, wtTDP-43 and TDP-43^ΔNLS^ show an increase in the presence of cytoplasmic misfolded SOD1, as compared to cells incubated with conditioned media from cells transfected with empty vector control and wtFUS. Scale bar: 75 μm.

Earlier work has established that aggregates composed of ALS-causing SOD1 mutants can nucleate aggregation of the same soluble mutant SOD1 protein *in vitro* and in *vivo*^21^,^22^, and that induction of HuWtSOD1 misfolding via transient transfection with mutant SOD1 triggers the propagation of HuWtSOD1 misfolding for at least 5 passages^19^. To test if conditioned media from TDP-43 and FUS-transfected HEK293 cells can transmit HuWtSOD1 misfolding, we first utilized primary spinal cord cultures containing motor neurons, astrocytes and microglia from transgenic E12-E14 mice over-expressing HuWtSOD1. These transgenic animals were used, as mouse wild-type SOD1 does not participate in propagated protein misfolding^19^, which is due to the lack of a key tryptophan residue at codon 32^18^. We used mixed spinal cord cultures as ALS is a non-cell autonomous disease, in which neuronal and non-neuronal cells of the nervous system participate in pathogenesis^23^. For the immunodetection of misfolded HuWtSOD1, we used a mouse monoclonal misfolded SOD1-specific antibody (3H1), generated against an extended electrostatic loop in misfolded SOD1 that is not antibody-accessible in its wild-type native conformation^18^. To induce misfolding of HuWtSOD1 in transfected cells, we used the naturally occurring R495x and P525L mutations of FUS, and an experimentally designed TDP-43 mutation with a dysfunctional nuclear localization signal (ΔNLS)^4^; exogenous TDP-43 and FUS were fused to HA-tag at their amino terminus. Conditioned media from the transfected cells was collected and concentrated, and overlaid on the primary cultures at 7 DIV (Supplementary Fig. 1). Our results show that primary spinal cord cultures incubated for 20 h with neural growth media containing the pellet fraction of HEK293-conditioned media from FUS^R495x^, FUS^P525L^, wtTDP-43 and TDP-43^ΔNLS^, but not empty vector control or wtFUS transfected cells, display significant neurite immunoreactivity for misfolded SOD1 by immunocytochemistry (Fig. 1), despite similar transfection efficiency of the various constructs in HEK293 cells (Supplementary Fig. 2). Background staining observed in primary cultures incubated with conditioned media from empty vector or wtFUS transfected cells could be attributed to newly translated HuWtSOD1, which may take hours to properly fold, become metalated, disulphide-oxidized and dimerized^24^. Incubation of spinal cord cultures from the non-transgenic littermates with conditioned media from TDP-43 or FUS transfected cells does not induce misfolding of SOD1 (Supplementary Fig. 3). This finding is consistent with induction and intercellular propagation of HuWtSOD1 misfolding by the TDP-43 or FUS-induced misfolded HuWtSOD1.

**Figure 2:**
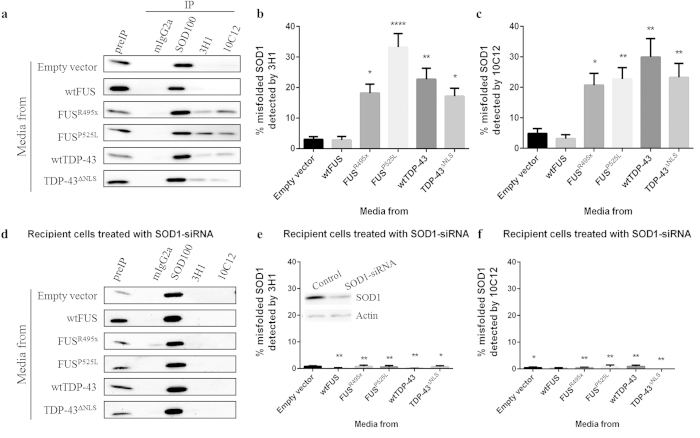
Propagation of misfolded HuWtSOD1 depends on endogenous HuWtSOD1 substrate. Representative immunoblots of quantitative immunoprecipitation of SOD1 proteins from untransfected (**a**) or SOD1-siRNA transfected (**d**) HEK293 cells incubated for 20 h with conditioned media from TDP-43 or FUS-transfected HEK293 cells. Immunoprecipitation studies were performed using a rabbit polyclonal pan-SOD1 antibody, SOD100, and misfolded SOD1-specific mouse monoclonal antibodies, 3H1 and 10C12. Mouse IgG2a isotype control was used as negative control, and blots were probed with pan-SOD1 antibody. Lysate pull-down signals from 3H1 (**b**) and 10C12 (**c**) were normalized to total immunoprecipitable SOD1 in each lysate and expressed as a percentage of total SOD1. We used non parametric one-way ANOVA to established statistical significance between cells incubated with conditioned media from FUS^R495x^, FUS^P525L^, wtTDP-43 and TDP-43^ΔNLS^ transfected cells as compared to cells incubated with conditioned media from empty vector transfected cells. Two tailed Student’s t-test was used to demonstrate statistically significant reduction in detectable misfolded SOD1 between SOD1-siRNA treated (**e**, **f**) and the corresponding untreated (**b**, **c**) recipient cell cultures incubated with the same conditioned media. Inset in (**d**) confirms downregulation of SOD1 using an immunoblot probed with pan-SOD1 and actin (load control) antibodies.*,p < 0.05 ; **,p < 0.01; ****,p < 0.0001. Number of biological repeats was 9 for empty vector and wtFUS, and 16 for the other constructs.

To measure the extent of HuWtSOD1 transmitted misfolding, we performed quantitative immunoprecipitations on human HEK293 cells that were incubated with conditioned media collected from TDP-43 or FUS transfected HEK293 cells. Lysates of recipient cells were immunoprecipitated using magnetic beads coupled to misfolded SOD1-specific antibodies, 3H1 and 10C12 (Fig. 2a). The latter antibody is a mouse monoclonal misfolded SOD1-specific antibody raised against an oxidized epitope within the SOD1 dimer interface, which is normally buried within the native SOD1 homodimer^4^,^18^. Consistent with the immunocytochemical observations in primary spinal cord cultures, 20–40% of the total immunoprecipitable HuWtSOD1 in HEK293 lysates was misfolded in cells that had been incubated with media from FUS^R495x^, FUS^P525L^, wtTDP-43 or TDP-43^ΔNLS^ transfected cells, but not empty vector control or wtFUS conditioned media (Fig. 2b,c).

To rule out the possibility that the immunoprecipitation signal in recipient HEK293 cells was merely due to passive uptake of misfolded HuWtSOD1 from conditioned media, we selectively knocked-down endogenous SOD1 in the recipient cells using SOD1-siRNA (Fig. 2e inset) prior to incubating recipient cells with conditioned media for 20 h (Fig. 2d). Quantitative immunoprecipitation demonstrated that SOD1-knockdown HEK293 cultures show a highly significant 15–25 fold drop in misfolded HuWtSOD1 compared to non-knockdown HEK293 cells when incubated with conditioned media originating from mutant FUS, as well as wild-type and mutant TDP-43, transfected cells (Fig. 2e,f). Immunoblots in Fig. 2d were transformed to clearly demonstrate the lack of immunorprecipitatable misfolded SOD1 in cultures with downregulated endogenous levels of SOD1. The ability of SOD1-siRNA to significantly reduce detectable misfolded SOD1 in recipient cells is consistent with the requirement of endogenous HuWtSOD1 as substrate for a prion-like conformational conversion process.

We also sought to determine if seeded conversion of HuWtSOD1 might prove to be toxic. Recipient cells incubated for 20 h with conditioned media from TDP-43 or FUS transfected cells were analyzed for viability using the MTT assay (Supplementary Fig. 4). Our results show that there is a modest but statistically significant reduction in cell viability in those cells incubated with conditioned media originating from FUS^R495x^, FUS^P525L^, wtTDP-43 or TDP-43^ΔNLS^ transfected cells. We used this data to estimate cell death to be between 13–15% when the conditioned media originated from misfolded-SOD1 producing cells, when compared to empty vector control.

### SOD1 misfolding-specific antibodies inhibit propagated misfolding of HuWtSOD1

**Figure 3:**
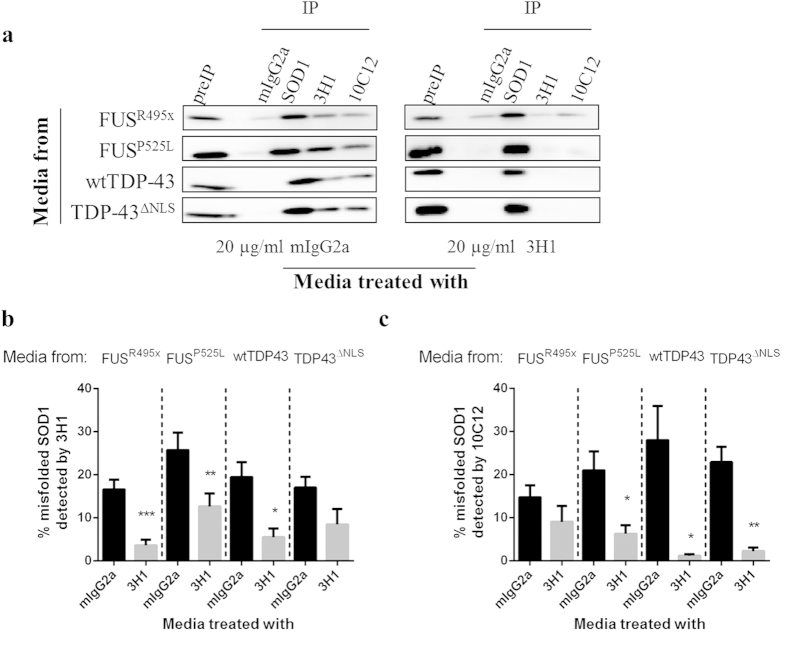
Intercellular propagation of HuWtSOD1 misfolding is inhibited by misfolded SOD1-specific antibodies. (**a**) Representative immunoblots of SOD1 immunoprecipitations from recipient cells incubated for 20 h with conditioned media that were pre-incubated with either 20 μg/ml 3H1 or mIgG2a isotype control. Immunoprecipitation studies were performed using pan-SOD1 antibody (SOD100), and misfolded SOD1-specific mouse monoclonal antibodies, 3H1 and 10C12. Blots were probed with pan-SOD1 antibody. Immunoprecipitation using 3H1 (**b**) and 10C12 (**c**) were quantified and expressed as a fraction of total immunoprecipitable SOD1 in each lysate. Statistical significance between the ability of 3H1and isotype control to inhibit induction of SOD1 misfolding for specified conditioned media was established using a two-tailed Student’s *t*-test (*,p < 0.05; **,p < 0.01; ***,p < 0.001). Graph bars represent 12 biological repeats per construct.

We previously established that pre-incubation of conditioned media from SOD1^G85R^ or SOD1^G127x^ -transfected cells with either misfolding-specific or pan-SOD1 antibodies inhibits transmission of propagated HuWtSOD1 misfolding *in vitro*^19^. To test the activity of 3H1, a potent inhibitor of propagated misfolding^19^, in TDP-43 and FUS-induced HuWtSOD1 intercellular transmission, conditioned media derived from HEK293 cells transfected with FUS^R495x^, FUS^P525L^, wtTDP-43 or TDP-43^ΔNLS^ were pre-incubated with 20 μg/ml of 3H1 antibody or mouse IgG2a isotype control (Fig. 3a). A significant reduction of 50–80% misfolded HuWtSOD1 was observed in HEK293 lysates incubated with 3H1-treated conditioned medium when analyzed by quantitative immunoprecipitation using 3H1 and 10C12, as compared to conditioned medium incubated with an isotype control (Fig. 3b,c). Although the neutralization effect is not complete, the efficiency is comparable to the level of inhibition of intercellular transmission of mutant SOD1-induced misfolded HuWtSOD1 using 3H1 19. Additionally, the ability of misfolded SOD1 specific antibodies to block transmission of protein misfolding is indicative of the presence of electrostatic loop-exposed misfolded SOD1 seed in the respective conditioned media.

### No propagation of TDP-43 or FUS pathology to recipient cells in the timeframe of SOD1 misfolding

**Figure 4:**
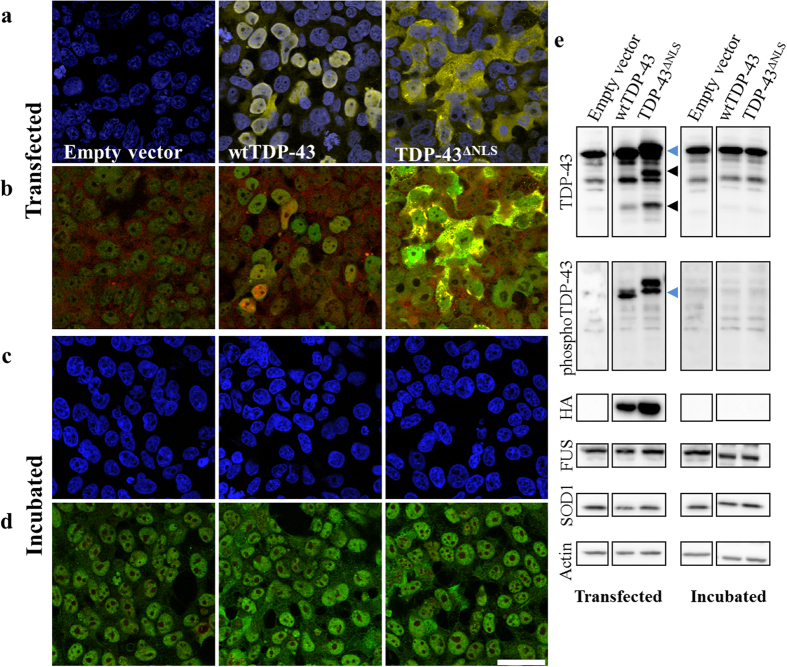
No intercellular propagation of TDP-43 pathology is detected in recipient cell cultures. Immunofluorescent staining of HEK293 cells transfected with TDP-43 using HA tag (yellow, **a**), or pan-TDP-43 (green, **b**) and phosphorylated-TDP-43 (red, **b**) antibodies shows an increase in TDP-43 mislocalization, cytoplasmic accumulation, and hyperphosphorylation (yellow punctae in ‘**b**’ correspond to colocalization). Pathological forms of TDP-43 are detected in significantly greater amounts when cells are transfected with TDP-43^ΔNLS^, but can be also detected with wtTDP-43. Immunofluorescent staining of HEK293 cells incubated for 20 h with conditioned media from TDP-43 transfected cells shows no signs of transmission of transfected (**c**) or pathological (**d**) TDP-43. In immunoblotting analysis of cell lysates, fragmentation (black arrowheads), hyperphosphorylation (blue arrowheads) and expression of exogenous TDP-43 are all present in the transfected, but not incubated cells (**e**). Abundance of FUS and SOD1 is not affected in either transfected or incubated cells, when compared to actin load control. Empty vector and TDP-43 panels for each antibody are cut-outs from the same gel. Scale bar: 50 μm.

**Figure 5:**
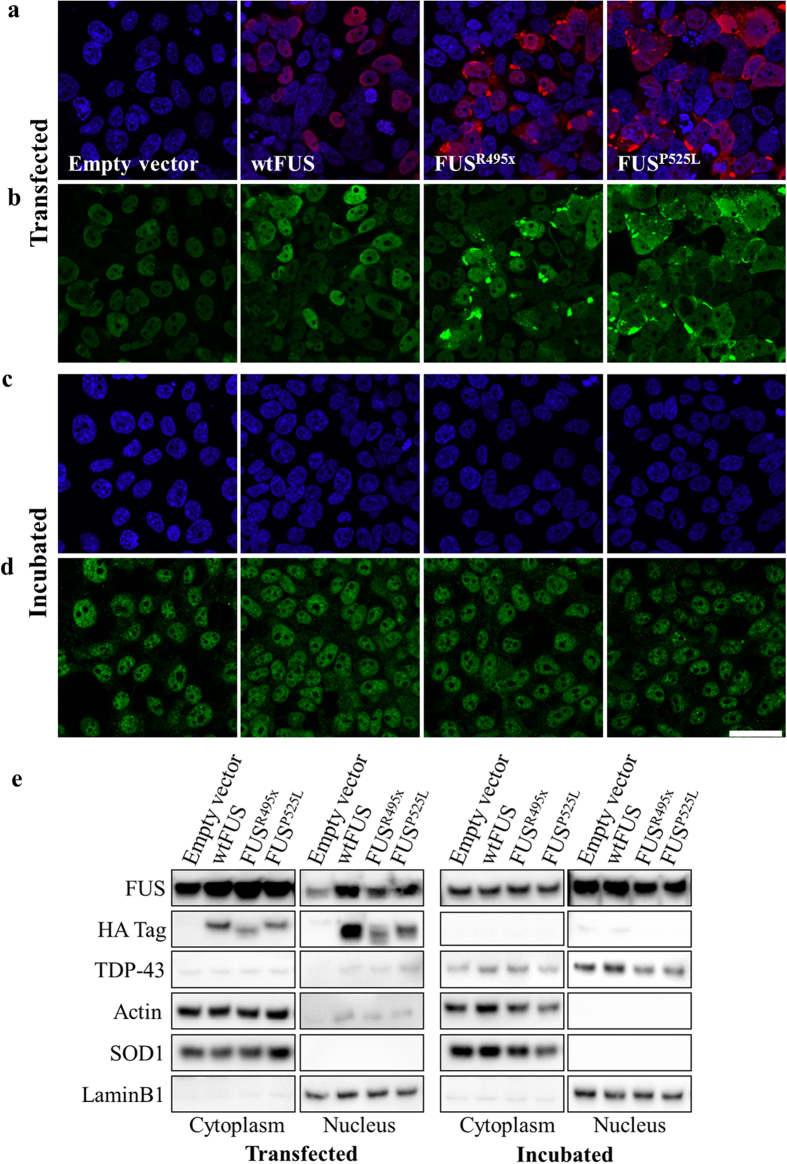
FUS pathology does not propagate between cell cultures through conditioned media. Immunofluorescent staining of HEK293 cells transfected with wtFUS, FUS^R495x^ or FUS^P525L^ using HA tag (red, **a**) or pan-FUS (green, **b**) antibodies shows mislocalization and aggregation of the exogenous FUS mutants. Cells transfected with mutant FUS contain cytoplasmic, and often aggregated protein, while exogenous wtFUS localizes in the nucleus. A similar staining performed on recipient HEK293 cell cultures that were incubated for 20 h with conditioned media from FUS transfected cells reveals no transmission of exogenous protein (**c**) or of pathology as can be seen by the lack of mislocalized or aggregated FUS (**d**) in the recipient cells. Cells were counterstained with Hoechst33342 (blue). Additionally, cells transfected with empty vector control, wild-type or mutant FUS, and cells incubated with conditioned media from cells transfected with the indicated construct, were fractionated into cytoplasmic and nuclear fractions (**e**). Actin was used as loading control, while LaminB1 and SOD1 were used as nuclear and cytoplasmic purity controls, respectively. Scale bar: 50 μm.

We used our HEK293 cell culture model (Supplementary Fig. 1), which supports the propagated misfolding of HuWtSOD1, to study the potential propagation of TDP-43 (Fig. 4) or FUS (Fig. 5) pathology from transfected cells to untreated recipient cells in the same experimental timeframe as propagated-misfolding of SOD1 occurs. Consistent with other studies, we confirmed pathological TDP-43 in transiently transfected HEK293 cells to be hyperphosphorylated, mislocalized and fragmented^9^ (Fig. 4a,b). While transient over-expression of wtTDP-43 is predominantly localized to the nucleus with several cytoplasmic punctae, transfection of the mutant TDP-43^ΔNLS^ results in nearly exclusive cytoplasmic localization and aggregation (Fig. 4a,b). Transfection of an empty vector control does not alter the predominantly nuclear localization of TDP-43. Immunofluorescent staining using a phospho-TDP-43 antibody reveals hyperphosphorylation of TDP-43 exclusively in cytoplasmic inclusions in HEK293 cells transfected with TDP-43^ΔNLS^, and to a significantly lesser extent with wtTDP-43 (Fig. 4b). However, these TDP-43 phenotypic features were not transmitted to recipient cell cultures following a 20 h incubation with conditioned media; neither pan-TDP-43 nor phospho-TDP-43 antibodies detected mislocalized or hyperphosphorylated TDP-43, respectively, in recipient cells following a 20 h incubation with conditioned media (HEK293 cells: Fig. 4c,d; Primary mouse spinal cord cultures: Supplementary Fig. 5). Immunoblotting analysis (Fig. 4e) confirmed our immunocytochemistry findings; HEK293 transfection of wild-type and mutant TDP-43, but not empty vector, results in: (1) hyperphosphorylation of full size TDP-43 (blue arrowheads); and (2) partial fragmentation of TDP-43 into 35 kDa and 25 kDa bands (black arrowheads). These signs of pathological TDP-43 are not observed in recipient cell cultures when incubated with conditioned media. Additionally, probing the immunoblots using an HA-tag antibody shows significant protein expression in cells transfected with TDP-43 constructs, but no expression in the incubated cells, indicating that the conditioned media contains no active residual lipofectamine reagent and that the transfection-encoded TDP-43 protein does not transmit to recipient HEK293 cell cultures (Fig. 4a,c,e).

Furthermore, we found that while mutant FUS is predominantly localized to the cytoplasm of transiently transfected HEK293 cells (Fig. 5a,b), this pathology is not transmitted to untreated recipient cell cultures when incubated with conditioned media from FUS^R495x^ or FUS^P525L^ transfected cells (Fig. 5c,d), in the same timeframe as misfolding of SOD1 occurs. Transfected wild-type FUS localizes predominantly in the nucleus, and does not trigger FUS mislocalization or aggregation in incubated cells (Fig. 5a,b). Cell fractionation into cytoplasmic and nuclear fractions (Fig. 5e) shows that transfection of HEK293 cells with wild-type or mutant FUS drives expression of the construct-encoded proteins in both nucleus and cytoplasm (although immunocytochemistry reveals cytoplasmic aggregates only with mutant FUS; Fig. 5a,b), while no exogenous protein is detectable in either fraction in cultures incubated with the indicated conditioned media (Fig. 5c–e). Furthermore, immunofluorescence and cell fractionation of recipient cells reveals no increase in cytoplasmic, or decrease in nuclear, endogenous FUS, indicative of absence of transmission of pathological FUS mislocalization *in vitro*. Expression levels and localization of TDP-43 and FUS are not affected by the incubation of cells with conditioned media. Notably, mutations in either TDP-43 or FUS can be found in ALS, but only wtTDP-43 pathology is observed in sporadic ALS 14; we find in the present study that pathological wtTDP-43, but not wtFUS, is associated with propagation-competent SOD1 misfolding.

## Discussion

An increasing body of evidence supports the notion that neurodegenerative disorders, including Alzheimer and Parkinson diseases, and ALS, can spread between contiguous and projection regions via the intercellular transmission of aggregated misfolded proteins and/or extracellular vesicles^21^,^25^,^26^,^27^,^28^. Previous reports have shown that aggregates composed of ALS-causing SOD1 mutants are taken up by cells, where they trigger the nucleation and aggregation of soluble mutant SOD1^22^. Also, overexpression of mutant SOD1 in cells can kindle the misfolding of endogenous wild-type SOD1^18^, which can propagate intercellularly and trigger additional rounds of misfolding of wild-type SOD1 in recipient cells^19^. Although cytotoxic mutations in SOD1 represent only a fifth of familial ALS cases, we and others reported on the presence of misfolded SOD1 in sporadic ALS and non-SOD1 familial ALS^3^,^4^,^5^,^19^, potentially implicating misfolded SOD1 as a pathogenic molecule in all types of ALS. We previously reported that pathogenic FUS and TDP-43, the latter of which is present in all SALS in its wild-type isoform, as well as non-SOD1 and non-FUS FALS^14^, trigger the intracellular misfolding of HuWtSOD1 in the same cells^4^ potentially through physical interaction between the aberrantly mislocalized and aggregated TDP-43 or FUS and HuWtSOD1. Alternatively, the kindling of SOD1 misfolding could occur through indirect mechanisms. Disease-implicated forms of TDP-43 and FUS have been recently reported to be associated with mitochondrial impairment^29^,^30^, which could lead to free radical generation and subsequent SOD1 misfolding through protein oxidation^31^. Other potential indirect mechanisms of SOD1 misfolding might include titration of chaperone proteins, or bulk saturation of clearance mechanisms such as proteosomal or autophagic degradation; in this latter regard, pathological TDP-43 and FUS are cleared via the proteasome^32^, thus reducing efficient clearance of misfolded SOD1^33^.

Here, we show for the first time that TDP-43 or FUS-induced misfolded HuWtSOD1 acquires the prion-like property of intercellular transmissibility and induction of endogenous HuWtSOD1 misfolding in recipient cells. The intercellular transmission of human mutant or misfolded wild-type SOD1 is likely to occur through release of naked aggregates by dying cells, which are taken up by macropinocyotosis and can trigger seeded aggregation^19^,^22^,^34^, or through the release of disease-associated exosomes containing intraluminal and surface-associated misfolded SOD1^19^,^35^. Following uptake of exosomes from the extracellular environment, the release of misfolded SOD1 into the recipient cells might occur through direct fusion of exosomal membrane with the plasma membrane, or by intraluminal fusion of the exosomes with the endosomal membrane following endocytosis^36^. We further find that knockdown of SOD1 expression in recipient cells prior to incubation with conditioned media leads to significant inhibition in the propagation of misfolded HuWtSOD1, consistent with HuWtSOD1 being the seed and substrate for the propagation of misfolding. This finding is consistent with the fact that in SOD1^G93A^ and SOD1^G37R^ experimental mouse models of ALS, suppression of mutant human SOD1 synthesis using adeno-associating virus encoding small hairpin RNA against mutant SOD1 led to improved motor performance, as well as a delay in disease onset and progression^37^. Taken together, this work indicates that the propagation of SOD1 misfolding is an active and independent process, which can occur in the absence of TDP-43 or FUS pathology. Our observation that propagated misfolded SOD1 is toxic to mesenchymal HEK293 cells may also provide insight into possibly pertinent mechanisms of neurodegeneration in ALS. Although transmissible TDP-43 or FUS-induced misfolded SOD1 triggers only a 13–15% decrease in HEK293 viability, we expect toxicity to be more marked in the more vulnerable end-mitotic motor neurons degenerating in disease. The cytotoxicity of the soluble TDP-43 or FUS-induced misfolded SOD1 is also consistent with reports showing similar toxicity of soluble mutant SOD1 to CHO cells^38^.

Familial, and to a significantly lesser degree sporadic, ALS can be partially explained by the presence of pathological mutations in a variety of proteins; recent research has shown that heritable mutations in some of these genes, for instance C9ORF72^39^, VCP^40^, VAPB^41^, and ataxin 2^42^, are linked to the pathophysiological hallmark of abnormal cytoplasmic wtTDP-43 accumulation in disease. Other disease-implicated proteins that are essential for proteostasis, such as P62^43^, ubiquilin 2^44^, and optineurin^45^, are also known modifiers of TDP-43 aggregation. Since misfolded SOD1 is detectable in both sporadic and familial ALS, including in spinal cords of patients with the C9ORF72 expansion mutation^19^, we now propose that in all SALS, non-SOD1 and non-FUS FALS, pathological aggregation of wtTDP-43 triggers a prion-like cycle of propagated misfolding of HuWtSOD1^4^.

Given reports that TDP-43 may act as a prion-like protein by inducing its own nucleation, mislocalization and propagation^17^,^46^, could HuWtSOD1 misfolding be secondary to TDP-43 or FUS pathology? Here, we provide a system to disentangle the relative contribution of TDP-43 or FUS and HuWtSOD1 to the prion-like intercellular transmission of propagated protein misfolding in ALS. Importantly, in this model, the recipient cell cultures were not primed to accept protein pathology by overexpressing the relevant proteins; rather, recipient cells expressed normal loads of endogenous SOD1, TDP-43 and FUS. We find that during the timeframe in which HuWtSOD1 misfolding propagates to recipient cells, no pathological TDP-43 or FUS is transmitted, indicating that spread of TDP-43 or FUS pathology is not necessary for the transmission of HuWtSOD1 misfolding. Neuronal cytoplasmic TDP-43 aggregation is also observed in non-ALS disorders such as Alzheimer’s disease^47^, where it is clearly not sufficient to induce the ALS syndrome. Even in frontotemporal dementia (FTD) associated with C9ORF72 mutation, TDP-43 pathology can be observed in spinal motor neurons without evidence of motor neuron cell death^48^, suggesting the existence of a molecular “second hit,” which could be the induction of propagated misfolding of HuWtSOD1. Moreover, transgenic mouse models expressing mutant and wtTDP-43 frequently do not exhibit the robust motor neuron disease observed in transgenic human mutant SOD1 mice, or in SOD1-FALS patients^49^. Notably, HuWtSOD1, which can trigger an ALS-like syndrome in mice, is completely competent to engage in propagated misfolding with itself, but is unable to do so with mouse SOD1 substrate^18^,^19^,^50^. These finding suggest that humans possess a critical vulnerability to SOD1 misfolding propagation (such as Trp32^18^) which is lacking in transgenic mouse models of TDP-43 or FUS pathology, supporting the idea that human SOD1 misfolding, induced by mutation or seeded propagation, is necessary and sufficient to cause the human ALS syndrome.

We report here that neutralization of the propagating TDP-43 or FUS-induced misfolded HuWtSOD1 using SOD1 misfolding-specific antibodies reduces the induction of protein misfolding by 50–80%, implying that misfolded HuWtSOD1 is a key participant in the intercellular transmission particle *in vitro*. Passive immunization of SOD1^G93A^ mice with anti-human SOD1 antibody, or other monoclonal antibodies specific to misfolded forms of SOD1, have been shown to reduce the burden of misfolded SOD1 in the spinal cord and prolong the survival of antibody-treated mice^51^,^52^. Furthermore, active immunization using SOD1 exposed dimer interface (SEDI) peptide in SOD1^G37R^ transgenic mice reduced the accumulation of misfolded SOD1 in the spinal cord, and increased survival by an average of 40 days^53^. Together, our data strengthens the viability of an immunological therapy approach against misfolded SOD1 in familial and sporadic ALS.

## Materials and Methods

### Cell culture

Human embryonic kidney cells (HEK293FT; ATCC, Manassas, VA) were cultured in complete Dulbecco’s Modified Eagle Medium (DMEM) containing 10% FBS, 10 U/ml penicillin, 10 U/ml streptomycin and 2 mM L-glutamine (Life Technologies, Carlsbad, CA). For immunoprecipitation and immunofluorescence studies, recipient cells were grown in 10 cm and 24-well tissue culture-treated plates, respectively. Cells were transiently transfected with TDP-43 or FUS plasmid DNA using Lipofectamine LTX (Life Technologies, Carlsbad, CA), according to manufacturer’s instructions. For the transfection of SOD1-siRNA, RNAiMaX reagent (Life Technologies, Carlsbad, CA) was used according to manufacturer’s instructions. Biological repeats refer to experiments that were performed on different weeks with cells of different passage. Cell handling, transfections, media transfer and biochemical analyses were also performed separately for each experiment to ensure true biological repeat.

### Preparation of conditioned media and antibody blocking

For induction of SOD1 misfolding in HEK293 cells, 48 h post transfection, conditioned media was collected from the transfected cells, and centrifuged at 1,000 × g for 5 min to remove floating cells and cell debris from the media. Clarified conditioned media were then supplemented with 25% of fresh complete DMEM media, and placed onto recipient cells for a 20 h incubation. For antibody blocking experiments, the conditioned media was prepared as described above, and subsequently incubated with 20 μg/ml of 3H1 or mIgG2a isotype control for 30 min at 37 °C with constant rotation, prior to adding it to recipient cells. For induction of human SOD1 misfolding in primary spinal cord cultures, competent misfolded SOD1 ‘seed’ was concentrated via ultracentrifugation at 100,000 × g for 1 h, as we previously determined that the majority of misfolded SOD1 is present in the pellet fraction of ultracentrifuged media^19^. The isolation was performed to minimize the transfer of DMEM growth media to the primary cultures that are cultured in complete Neurobasal media (Life Technologies, Carlsbad, CA; supplemented with neuronal supplements [StemCell Technologies, Vancouver, BC], 10 U/ml penicillin, 10 U/ml streptomycin and 2 mM L-glutamine). Pellets were resuspended in complete Neurobasal media, and added to spinal cord cultures for a 20 h incubation.

### Immunoprecipitation

Cells incubated with conditioned media were grown on 10 cm tissue culture-treated plates, and lysed in 400 μl of lysis buffer (0.5% Triton X-100, 0.5% sodium deoxycholate in PBS, and EDTA-free protease inhibitors, pH 7.4). For immunoprecipitation experiments, 100 μl of cell lysate was mixed with 10 μl of antibody-coupled M-280 Tosyl-activated magnetic Dynabeads^4^ (Life Technologies, Carlsbad, CA). Tubes were incubated for 3 h at room temperature with constant rotation. Beads were then washed 3 times with 150 μl of RIPA buffer (150 mM NaCl, 50 mM Tris-HCl, pH 8.0, 1% Nonidet P-40, 0.5% sodium deoxycholate, 0.1% SDS) with brief agitation between washes, and boiled in loading sample buffer containing 1% β-mercaptoethanol. Pre-IP consists of 1 μl lysate boiled in sample buffer.

## Additional Information

**How to cite this article**: Pokrishevsky, E. *et al.* TDP-43 or FUS-induced misfolded human wild-type SOD1 can propagate intercellularly in a prion-like fashion. *Sci. Rep.* **6**, 22155; doi: 10.1038/srep22155 (2016).

## Supplementary Material

Supplementary Information
