# Supplementary material for: TDP-43 or FUS-induced misfolded human wild-type SOD1 can propagate intercellularly in a prion-like fashion
Source: Sci Rep. 2016 Mar 1;6:22155. doi: 10.1038/srep22155 (PMC4772009; doi:10.1038/srep22155)
Supplement: Supplementary Information [file srep22155-s1.pdf]

Supplementary Information

TDP-43 and FUS-induced misfolded human wild-type SOD1 can propagate intercellularly in a prion-like fashion

Edward Pokrishevsky, Leslie I Grad, Neil R Cashman

Supplementary Materials and Methods

Immunocytochemistry

Cells were placed on glass cover-slips (#1.5) in a 24 well plate prior to transfection or incubation with conditioned media. For immunofluorescence study, cells were washed twice with ice-cold phosphate buffer saline (PBS) and fixed in 4% paraformaldehyde (in PBS, pH 7.4) for 15 min at room temperature. Fixed cells were then washed once with PBS, permeabilized for 10 min using PBSTx (0.3% Triton X-100 in PBS), and blocked for 30 min with incubation buffer (2% normal goat serum in PBS, filtered). Cells were then incubated with the following primary antibodies: 2 µg/ml anti SOD1 misfolding-specific mouse monoclonal 3H1 [1,2], 1 µg/ml rat anti HA tag (Roche Diagnostics, IN), 10 µg/ml rabbit anti TDP-43 (ProteinTech Group Inc., Chicago, IL), 10 µg/ml rabbit anti phosphor-(409/410) TDP-43 (ProteinTech Group Inc., Chicago, IL), 10 µg/ml mouse anti FUS (ProteinTech Group Inc., Chicago, IL) diluted in incubation buffer for 1 h at room temperature. Cell were then washed twice in PBS, and incubated with appropriate secondary antibody conjugated to Alexa Fluor-488 or 647 fluorescent dyes (Life Technologies, Carlsbad, CA; 1:1000 dilution) for 1 h at room temperature in the dark. The cells were washed with PBS, and DNA was counterstained using 2 µg/ml Bis-benzimide H33342 trihydrochloride (Hoechst 33342) for 5 min. Following 2 final washes the cells were mounted on a glass slide in a drop of Fluoromount-G (SouthernBiotech, Birmingham, AL). Confocal images were captured using Leica TCS SP8 microscope (Leica Canada) using the LAS-X software. Images were acquired at the same settings and not subjected to any further image processing.

Immunoblotting and quantification

Cell lysates were prepared by incubating pelleted cells with a mild lysis buffer containing 0.5% sodium deoxycholate, 0.5% Triton X-100 and EDTA-free protease inhibitor cocktail (in PBS; for analysis using phosphoTDP-43, phosphatase inhibitors were added) for 2 min on ice, followed by centrifugation for 5 min at 1,000 x g at 4℃. Prepared samples were boiled in sample buffer containing 1% β-mercaptoethanol, and analyzed on 4-20% Tris-Glycine gels (LifeTechnologies, Carlsbad, CA). Proteins were then transferred to a PVDV membrane, blocked (Tris-buffer saline, 0.1% Tween-20 (TBST) with 3% BSA for phosphor-(409/410) TDP-43 or 5% skimmed milk for all other proteins) for 1 h , and incubated with 1 µg/ml SOD100 (Assay Designs, Ann Arbor, MI), 10 µg/ml

TDP-43, 10 µg/ml phosphor-(409/410) TDP-43, 10 µg/ml FUS (Abcam, Cambridge, MA), 5 anti-HA (Abcam, Cambridge, MA), 0.5 µg/ml of Lamin B1(Abcam, Cambridge, MA), or 1 µg/ml actin (ABM Inc., Richmond, BC) antibodies overnight at 4°C. Membranes were then washed twice in TBST and incubated with anti-mouse or rabbit IgG horseradish peroxidase linked whole antibody (GE Healthcare, Buckinghamshire, UK) diluted 1:10,000 in blocking buffer for 1 h at room temperature. Membranes were developed using SuperSignal West Femto chemiluminescence susbtrate (Thermo Scientific, Waltham, MA), and visualized using VersaDoc Imager (Bio-Rad Laboratories, Hercules, CA) with no digital or biological signal saturation. The densitometry of western blot bands was quantified using Quantity One software. Percentage of misfolded SOD1 was calculated by quantifying specific immunoprecipitation of 3H1 or 10C12 using densitometry, subtracting pull-down with mIgG2a isotype control, and normalizing to total immunoprecipitable SOD1.

Nuclear and cytoplasmic extraction

Cells were grown in 6 cm plates one day prior to either transfection for 48 h or incubation for 24 h with conditioned media. On collection day, cells were washed twice in cold PBS, collected in a microcentrifuge tube, and spun for 5 min at 1,000 x g. Cell pellets were lysed on ice in cytoplamic extract (CE) buffer (10 mM HEPES, 10 Mm KCl, 10 mM EDTA, 1.5 mM $MgCl_2$, 0.4% NP40, 1mM DTT and protease inhibitors; adjusted to pH 7.9 and filtered), and centrifuged for 5 min at 14,000 x g. Supernatants were removed to fresh tubes and centrifuged for an additional 10 min, resulting in cytoplasmic fractions. Nuclei containing pellet were washed once in buffer CE, and incubated in nuclear extract (NE) buffer (20 mM HEPES, 400 mM NaCl, 1 mM EDTA, 10% glycerol, 1 mM DTT and protease inhibitors; adjusted to pH 7.9 and filtered) on ice for 90 minutes with brief vortexing every 20 minutes. Mixtures were then centrifuged for 5 min at 14,000 x g, and supernatants containing nuclear fractions were transferred to fresh tubes.

Mouse Primary Spinal Cord Culture

Experiments involving animals were conducted according to the Canadian Council on Animal Care guidelines and have been approved by the Animal Care Committee of the University of British Columbia. Pregnant C57 BL/6 female mice (Strain: B6SJL-Tg(SOD1)2Gur/J, Stock:002297; Jackson Laboratories, Bar Harbor, ME) were sacrificed according to the guidelines of the Institutional Animal Care and Use Committee (IACUC). Primary spinal

cord cultures were prepared from 12–14 day fetal mice using minor modification of an established protocol[3]. Following embryo genotyping for human wtSOD1, cervical, thoracic and lumbar- regions of the spinal cord were dissected out in Ca^{2+}/Mg^{2+}-free Hanks Balanced Salts (Life Technologies, Carlsbad, CA). Meninges were removed and the tissue was transferred to 0.25% trypsin (Life Technologies, Carlsbad, CA) and digested at 37°C for 15 min. Tissue was then resuspended in DMEM (Life Technologies, Carlsbad, CA) plus 10% fetal bovine serum (Life Technologies, Carlsbad, CA) and triturated 4–6 times through a fire-polished tip. The supernatant was centrifuged at 200 × g for 45 sec. Pelleted neural cells were resuspended in Neurobasal media, B27 supplements, 2 mM L-glutamine (all from Life Technologies, Carlsbad, CA) and seeded at a density of 2×10^5 cells/well onto poly-D-lysine (Sigma, Saint Louis, MO) coated #1.5 coverslips in 24-well plates. Cultures were maintained in serum-free Neurobasal-B27 medium, and one-half of medium was replaced on day 3 with equal volume of fresh medium. Cells were incubated with conditioned media at 7 DIV.

Cell Viability

Cells were grown in a 96-well plate and incubated overnight with conditioned media. Stock 3-(4,5-Dimethylthiazol-2-yl)-2,5-Diphenyltetrazolium Bromide (MTT; Sigma-Aldrich, MO) was prepared at 5 mg/ml in warm PBS and filtered, added directly to culture media to a final concentration of 0.5 mg/ml, and incubated for 3 h. Absorbance was read at 570 and 650 nm. For every biological MTT repeat, 4-8 technical repeats were performed.

Statistical Analysis

We tested every set of data for Gaussian distribution. For normal distributions we used the parametric one-way ANOVA test. Otherwise, without any assumption regarding the distribution underlying our sample sets, we applied nonparametric Kruskal-Wallis test. The significance thresholds were also adjusted for multiple comparisons by the Bonferroni correction to maintain the familywise error rate and keep the alpha level at 0.05.

Supplementary Figures

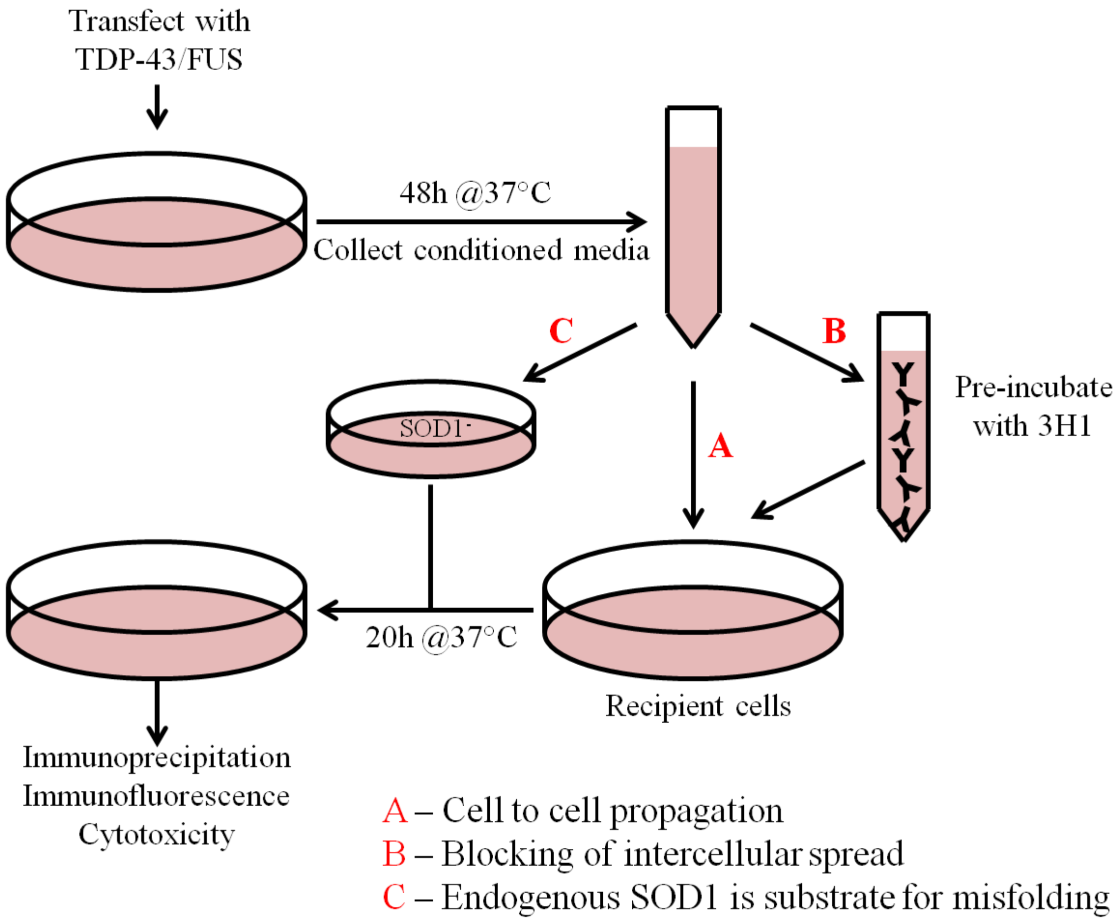

Supplementary Figure 1: Experimental flow chart. HEK293 cells were transfected for 48 h, following which the conditioned media was collected and pre-cleared. Afterwards, conditioned media was either placed for an additional 24 h on untreated recipient cells for determining the ability of TDP-43 or FUS-induced misfolded SOD1 to induce further rounds of HuWtSOD1 misfolding (**A**). In (**B**), conditioned media was pre-treated with a misfolding specific antibody, 3H1, prior to placement on fresh cell cultures. Alternatively, conditioned media was placed on SOD1-siRNA treated cells for determining whether HuWtSOD1 is an obligate substrate for misfolding (**C**).

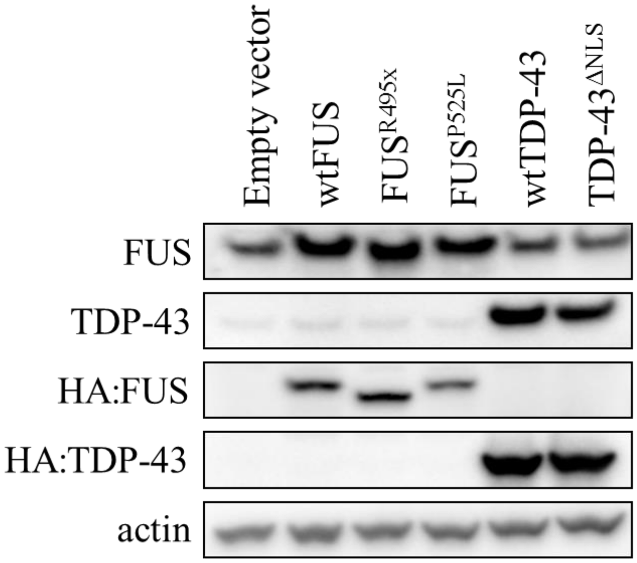

Supplementary Figure 2: Expression of pathological TDP-43 and FUS in HEK293 cells. Cell cultures were transfected with the indicated construct for 48 h, following which they were lysed using RIPA buffer (50mM Tris,150 mM NaCl, 0.1% SDS, 0.5% sodium deoxycholate, 1% NP-40, protease inhibitors in water) and analyzed for the presence of the exogenous protein. Immunoblots were probed using FUS or TDP-43 antibodies (top two rows) to visualize total proteins in lysates, or with HA-tag (middle two rows) for the detection of transfection - encoded proteins. Actin was used as loading control.

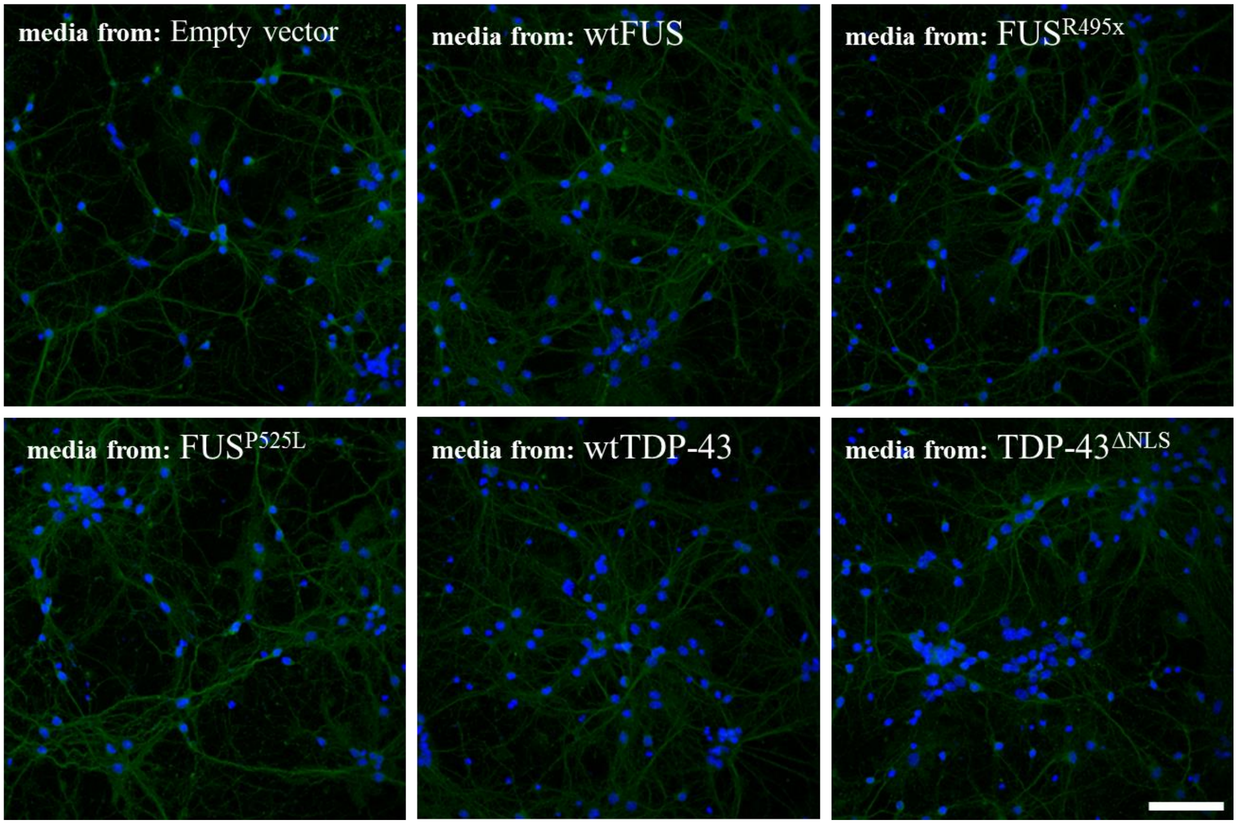

Supplementary Figure 3: TDP-43 and FUS-induced misfolded HuWtSOD1 does not kindle the misfolding of mouse SOD1. Primary spinal cord neural cultures prepared from wild-type mice were incubated for 20 h with conditioned media from transfected HEK293 cells, and stained for misfolded SOD1 (green) using misfolded SOD1-specific antibody 3H1 and counterstained using Hoechst 33342 (blue). The source of the media is indicated for each panel. Scale bar: 75 µm.

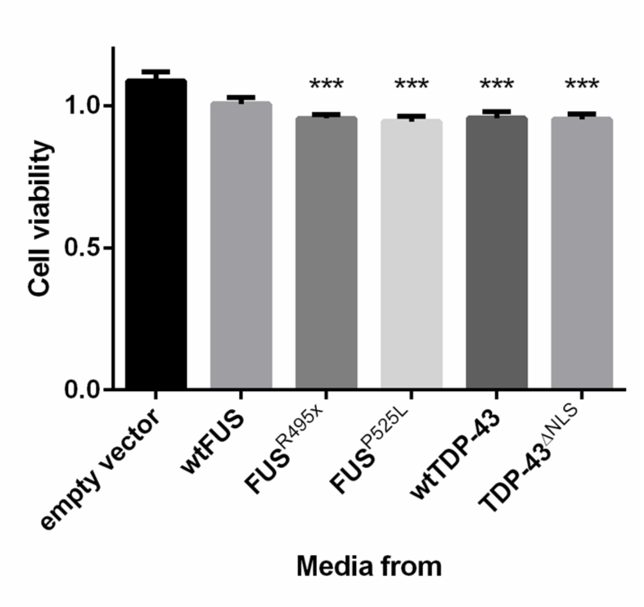

Supplementary Figure 4: Misfolded SOD1-containing media are cytotoxic to recipient HEK293 cells. Cell viability was determined by using a colorimetric assay, MTT, on recipient cells that were incubated with conditioned media from TDP-43 or FUS transfected cells for 20 h. When compared to cells incubated with conditioned media from cells transfected with empty vector control, a significant 13-15% reduction in cell viability (***, $p < 0.001$) is established in cells incubated with conditioned media from mutant FUS, as well as wild-type and mutant TDP-43, but not from wtFUS or empty vector transfected cells. Number of biological repeats is 13. Error bars represent s.e.m.

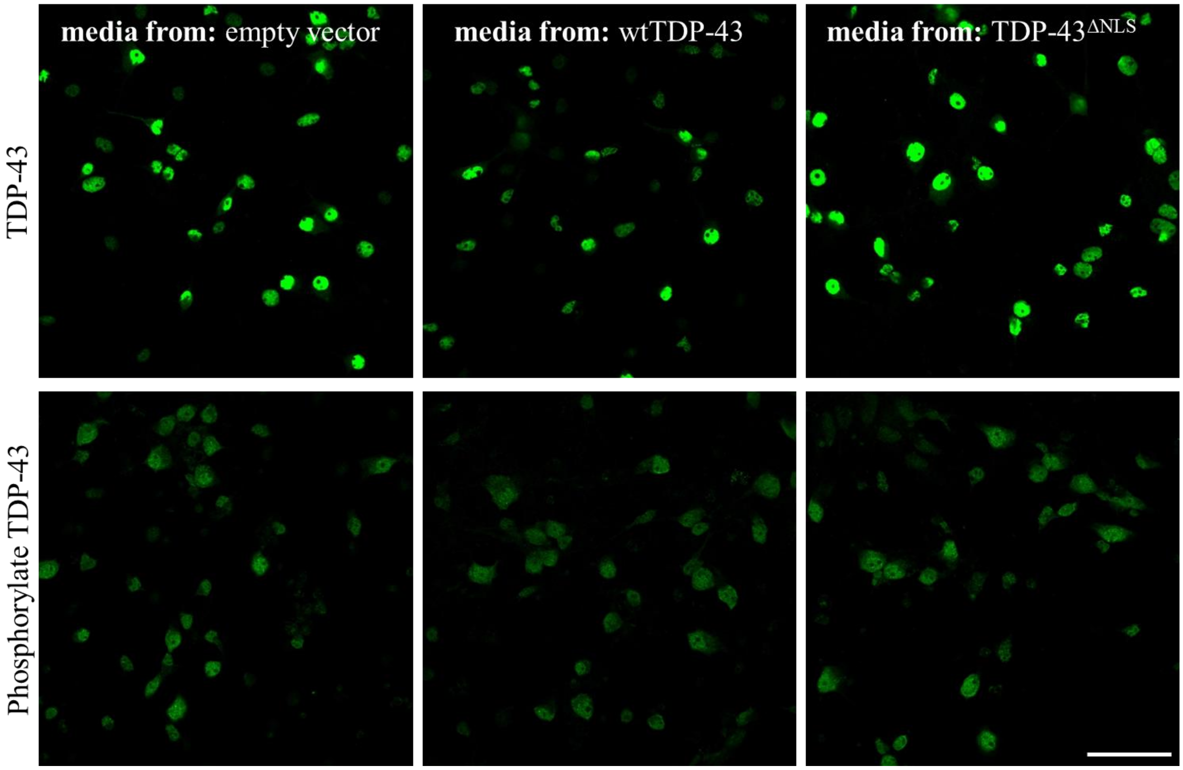

Supplementary Figure 5: No propagation of TDP-43 pathology to primary spinal cord cultures. Primary spinal cord cultures were incubated with conditioned media from empty vector, wild-type or mutant TDP-43 transfected cells. 24 h following incubation, cells were fixed and stained using pan (top; green) or P409/410 phosphoTDP-43 antibody (bottom; green). No mislocalization, aggregation or hyperphosphorylation of TDP-43 is detectable in these incubated cultures. Scale bar: 50 µm.

References

1. Pokrishevsky, E.*, et al.* Aberrant localization of FUS and TDP43 is associated with misfolding of SOD1 in amyotrophic lateral sclerosis. *PloS one* **7**, e35050 (2012).
2. Guest, W.C.*, et al.* Generalization of the prion hypothesis to other neurodegenerative diseases: an imperfect fit. *J Toxicol Environ Health A* **74**, 1433-1459 (2011).
3. Anderson, K.N.*, et al.* Isolation and culture of motor neurons from the newborn mouse spinal cord. *Brain research. Brain research protocols* **12**, 132-136 (2004).
